# Predictive Accuracy of 29-Comorbidity Index for In-Hospital Deaths in US Adult Hospitalizations with a Diagnosis of Venous Thromboembolism

**DOI:** 10.1371/journal.pone.0070061

**Published:** 2013-07-26

**Authors:** James Tsai, Karon Abe, Sheree L. Boulet, Michele G. Beckman, W. Craig Hooper, Althea M. Grant

**Affiliations:** 1 Division of Blood Disorders, National Center on Birth Defects and Developmental Disabilities Centers for Disease Control and Prevention, Atlanta, Georgia, United States of America; 2 Division of Reproductive Health, National Center for Chronic Disease Prevention and Health Promotion Centers for Disease Control and Prevention, Atlanta, Georgia, United States of America; Duke-NUS Graduate Medical School, Singapore

## Abstract

**Background:**

Venous thromboembolism (VTE), comprising deep vein thrombosis (DVT) and pulmonary embolism (PE), is a significant source of mortality and morbidity worldwide. By analyzing data of the 2010 Nationwide Inpatient Sample from the Agency for Healthcare Research and Quality (AHRQ), we evaluated the predictive accuracy of the AHRQ’s 29-comorbidity index with in-hospital death among US adult hospitalizations with a diagnosis of VTE.

**Methods:**

We assessed the case-fatality and prevalence of comorbidities among a sample of 153,518 adult hospitalizations with a diagnosis of VTE that comprised 87,605 DVTs and 65,913 PEs (with and without DVT). We estimated adjusted odds ratios and 95% confidence intervals with multivariable logistic regression models by using comorbidities as predictors and status of in-hospital death as an outcome variable. We assessed the c-statistics for the predictive accuracy of the logistic regression models.

**Results:**

In 2010, approximately 41,944 in-hospital deaths (20,212 with DVT and 21,732 with PE) occurred among 770,137 hospitalizations with a diagnosis of VTE. When compared separately to hospitalizations with VTE, DVT, or PE that had no corresponding comorbidities, congestive heart failure, chronic pulmonary disease, coagulopathy, liver disease, lymphoma, fluid and electrolyte disorders, metastatic cancer, other neurological disorders, peripheral vascular disorders, pulmonary circulation disorders, renal failure, solid tumor without metastasis, and weight loss were positively and independently associated with 10%−125% increased likelihoods of in-hospital death. The c-statistic values ranged from 0.776 to 0.802.

**Conclusion:**

The results of this study indicated that comorbidity was associated independently with risk of death among hospitalizations with VTE and among hospitalizations with DVT or PE. The AHRQ 29-comorbidity index provides acceptable to excellent predictive accuracy for in-hospital deaths among adult hospitalizations with VTE and among those with DVT or PE.

## Introduction

Venous thromboembolism (VTE), comprising deep vein thrombosis (DVT) and pulmonary embolism (PE), is a significant source of mortality, morbidity, and impaired health-related quality of life worldwide [1]–[4]. The *2008 Surgeon General’s Call to Action to Prevent Deep Vein Thrombosis and Pulmonary Embolism* suggested that VTE might be responsible for approximately 100,000−180,000 deaths per year in the United States [5]. Each year in the United States, there are more than a half-million adult hospitalizations with a diagnosis of VTE [6]. Available evidence suggests that a substantial portion of VTE events also occur among outpatients. Spencer et al. found 74% of patients with a confirmed episode of VTE had developed VTE in the outpatient setting [7]. However, more than a third of these outpatients with VTE might have had recent hospitalizations [7]. Furthermore, many deaths among hospitalizations with VTE might be attributable to concurrent VTE and other health conditions [8], [9]. Research has demonstrated that comorbidities can affect one or more diseases through several potential etiological mechanisms of direct causation, associated risk factors, heterogeneity, and independence [10]. Particularly among patients with VTE, the presence of comorbidities can hinder clinical assessment and timely diagnoses [11]–[13] and exacerbate the risk of recurrence, complications, and death [14]–[17].

Comorbidity indices are important tools to characterize disease burden and complexity, and to identify the prognostic factors that are essential for clinical assessments and decision-making [10], [18]–[20]. In observational studies that investigate the relationship between risk factors and health outcomes of interest, comorbidity indices frequently are used as a risk-adjustment to control for potential confounders [18]. Because the performance of individual comorbidity indices can vary, researchers often validate applicable indices by determining their ability to predict specific health outcomes, such as death among a target patient population [21]–[23]. Presently, surprisingly little research has been conducted to evaluate the performance of comorbidity indices for predicting death among patients with VTE. The Elixhauser Comorbidity Index, first reported in 1998, has been used increasingly in research with administrative data [24]–[26]. Although the original Elixhauser Comorbidity Index comprised 30 comorbidities based on the *International Classification of Diseases, Ninth Revision, Clinical Modification* (*ICD-9-CM*) coding algorithms for diagnoses and diagnosis-related groups, the Agency for Healthcare Research and Quality’s (AHRQ) Nationwide Inpatient Sample (NIS) included 29 of these comorbidity variables after excluding cardiac arrhythmias due to concerns about reliability [27]–[29]. To the best of our knowledge, the performance of the AHRQ 29-comorbidity index for predicting in-hospital death in adult hospitalizations in the United States with a diagnosis of VTE and among those with a diagnosis of DVT or PE has not been validated. Therefore, we sought to evaluate the predictive accuracy of the AHRQ 29-comorbidity index for in-hospital death among US adult hospitalizations with a diagnosis of VTE and among hospitalizations with a diagnosis of DVT or PE by analyzing data from the 2010 NIS.

## Methods

### Data Source

All procedures involving human participants and confidentiality were reviewed and approved by the Research Ethics Review Board of the AHRQ. Analysis of deidentified data from the NIS is exempt from the federal regulations for the protection of human research participants. The NIS is part of the Healthcare Cost and Utilization Project supported by the AHRQ. It is the largest all-payer inpatient care database in the United States with 5−8 million annual unweighted hospitalizations of patients covered by Medicare, Medicaid, or private insurance, and the uninsured from about 1,000 community hospitals. The NIS sampling frame consists of non-federal, short-term, general and specialty hospitals, and long-term acute care facilities. Excluded from the NIS are short-term rehabilitation hospitals, long-term non-acute care hospitals, psychiatric hospitals, and alcoholism or chemical dependency treatment facilities. The 2010 inpatient core file contained data for 7,800,441 hospitalizations drawn from 1,051 hospitals of participating states that make up 96% of the US population. The NIS is designed to approximate a 20% stratified sample of U.S. community hospitals that include all hospitalizations in sampled hospitals [30]. Details about the sampling methodology are described elsewhere [30].

### Sample of Hospitalization

The NIS has a maximum of 25 diagnostic codes based on the *ICD-9-CM* for each sampled hospitalization. VTE was identified using *ICD-9-CM* codes 415.1x, 451.1x, 451.2, 451.8x, 451.9, 453.2, 453.4x, 453.8x, and 453.9 in any of the diagnostic fields. We restricted our analysis to an unweighted sample of hospitalizations for adults 18 years of age or older with a diagnosis of VTE (n  = 154,785). Studies of comorbidities in adult hospitalizations often excluded maternal-related hospitalizations, because such patients were mostly young with low incidence of comorbidities and VTE and with a small risk of dying during hospital stay, even though the risk of developing VTE might be elevated during pregnancy and postpartum period [28], [31]–[33]. Therefore, after excluding hospitalizations due to pregnancy, childbirth, and puerperium and variables with missing information for sex, vital status, length of hospital stay, or status of primary expected payer, 153,518 unweighted hospitalizations with a diagnosis of VTE were included as the analytic sample, which comprised 87,605 hospitalizations with a DVT (DVT only) diagnosis and 65,913 hospitalizations with a PE (PE with and PE without DVT) diagnosis as separate subgroups. Hereinafter, subgroup of PE with or without DVT will be described as subgroup of PE.

### Demographic, Clinical, and Insurance Characteristics

The demographic variables included age, sex, and race or ethnicity (10.7% unstated). In addition, we included clinical characteristics and insurance status (i.e., total days of hospital stay, insurance status of primary expected payer, and status of operating room procedure) as covariates, because of their relevance to the outcome and prevention of VTE. All *ICD-9-CM* procedure codes in the NIS are assigned to one of four broad categories of minor diagnostic, minor therapeutic, major diagnostic, and major therapeutic procedures according to the *AHRQ Procedure Classes* [34]. An operating room procedure is defined as having at least one major diagnostic or major therapeutic procedure during hospitalization [35].

### AHRQ 29-Comorbidity Index

The 29 comorbidities of the NIS are considered as coexisting medical conditions that are not directly related to the principal diagnosis or the main reason for admission, and are likely to have existed prior to the hospital stay [27]. Each of the 29 comorbidities is based on a unique set of *ICD-9-CM* codes. Because the calculation of the category of pulmonary circulation disorders by the NIS comorbidity software (v3.7) included codes for PE as well as codes for other diseases in the category (e.g., pulmonary hypertension), we classified those hospitalizations with PE and without other disease in the category as having negative comorbidity status for pulmonary circulation disorders.

### Statistical Analysis

For adult hospitalizations with a diagnosis of VTE and hospitalizations with a diagnosis of DVT or PE, we estimated the number of in-hospital deaths and case-fatality rates that were stratified by age, sex, race/ethnicity, total days of hospital stay, insurance status of primary expected payer, status of operating room procedure, and number of comorbidities. In addition, we calculated the percentage distributions of in-hospital deaths by the number of comorbidities (each comorbidity was counted as 1) and assessed the prevalence of each of the 29 comorbidities for these three groups of hospitalizations. To determine the comorbidities that were associated independently with in-hospital death, we used a backward elimination procedure of stepwise regression to remove any explanatory variable with the highest *P*≥0.05 for individual *t*-test of null hypothesis β = 0. We repeated the same procedure until *P*<0.05 for all explanatory variables in the final model. Adjusted odds ratios (aORs) and 95% confidence intervals (CIs) for in-hospital death were generated by using multivariable logistic regression models to measure independent associations between remaining comorbidities and in-hospital death while controlling for age, sex, race or ethnicity, total days of hospital stay, insurance status of primary expected payer, and status of operating room procedure. The concordance statistic (or c-statistic), equivalent to the area under the receiver operating characteristic (ROC) curve, was calculated to assess the performance or discriminative power of logistic regression models in predicting death [36]–[38]. Compared to a randomly selected subject (i.e., hospitalization of patient) without the outcome (i.e., in-hospital death), the c-statistic was the probability that a randomly selected subject with the outcome correctly had been assigned a higher predicted probability of death by logistic regression model [37], [38]. In other words, among all possible pairs of hospitalizations consisting of one with the outcome (i.e., in-hospital death) and the other without the outcome, the c-statistic was the proportion of such pairs in which the hospitalization with the outcome received a higher predicted probability of death from the logistic regression model when compared with the hospitalization without the outcome [37]. The values of c-statistic can range from 0.5 (chance) to 1 (perfect). The values of 0.7 to <0.8, 0.8 to <0.9, and ≥0.9 indicate acceptable, excellent, and outstanding predictive accuracy, respectively [36]. The values of c-statistic were obtained from logistic regression and the ROC models that accounted for the sampling weights, stratification, and clustering of survey data by using the bootstrap replication estimation. We performed the data management and analysis using SPSS 21 Complex Samples for Survey Analysis (*IBM Corp*) and STATA 11 (*StataCorp LP*). Unless otherwise noted as an unweighted sample size (n), all estimates were weighted to account for the complex sampling design.

## Results

**Figure 1:**
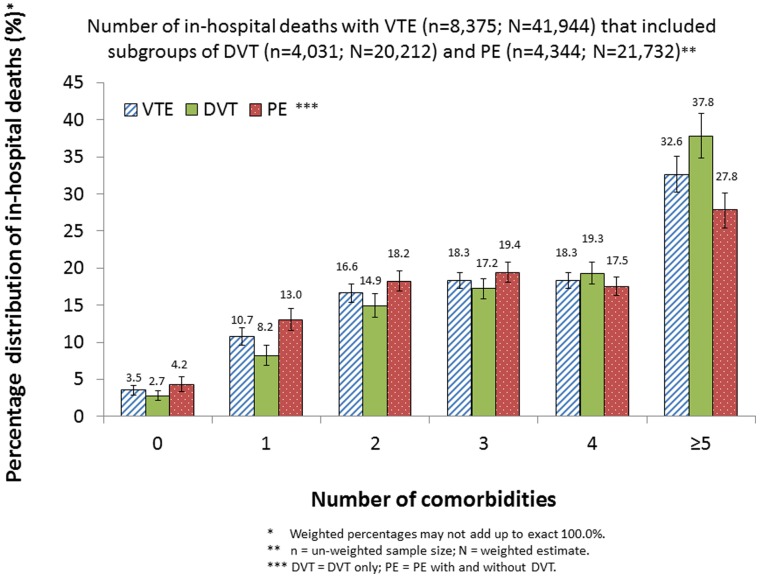
Estimated percentage distribution of in-hospital deaths with a diagnosis of VTE and among those with DVT or PE by number of comorbidity, NIS, 2010.

We found that a total of 41,944 in-hospital deaths occurred among 770,137 hospitalizations with a diagnosis of VTE in 2010, comprising 20,212 deaths among 440,151 hospitalizations with DVT and 21,732 deaths among 329,986 hospitalizations with PE. Correspondingly, the case-fatality rates were 5.4% for hospitalizations with VTE, and 4.6% and 6.6% for hospitalizations with DVT and PE, respectively. The case-fatality rates varied significantly by age, sex, race or ethnicity, total days of hospital stay, insurance status of primary expected payer, status of operating room procedure, and number of comorbidities (*P*<0.05) (Table 1). High case-fatality rates were observed among subgroups in which patients were 80 years of age or older, were male, were other race or ethnicity (included categories of “Asian or Pacific Islander,” “Native American,” and “other race/ethnicity”), had a hospital stay of at least 7 days, had Medicare as primary expected payer, had a “non-operating room procedure” and had at least five comorbidities (Table 1). While as many as 69.2% in-hospital deaths with a diagnosis of VTE had at least three comorbidities and 32.6% in-hospital deaths with a diagnosis of VTE had at least five comorbidities, similar proportions of distribution were found for in-hospital with DVT or PE (Figure 1).

**Table 1:** Case-fatality rates for in-hospital death among adult hospitalizations with a diagnosis of VTE and among those with DVT or PE by demographic and clinical characteristics, NIS, 2010.

Characteristics		Case-fatality rate for in-hospital death								
		VTE(n = 153,518; N = 770,137)			DVT^a^(n = 87,605; N = 440,151)^c^			PE^b^(n = 65,913;N = 329,986)		
	n	%	CI^d^	*P* e	%	CI	*P*	%	CI	*P*
**Overall**	153,518	5.4	5.2−5.7		4.6	4.3−4.9		6.6	6.3−6.9	
**Age**				*<0.001*			*<0.001*			*<0.001*
18−49	33,054	2.6	2.4−2.9		1.9	1.7−2.2		3.5	3.1−3.9	
50−79	88,128	5.8	5.5−6.0		4.9	4.6−5.3		6.8	6.5−7.1	
≥80	32,336	7.5	7.1−8.0		6.2	5.7−6.7		9.8	9.2−10.4	
**Sex**				* = 0.001*			* = 0.049*			*<0.001*
Male	73,592	5.6	5.4−5.9		4.7	4.4−5.1		6.9	6.5−7.2	
Female	79,926	5.3	5.0−5.5		4.5	4.2−4.8		6.3	6.0−6.7	
**Race/ethnicity** f				*<0.001*			* = 0.005*			*<0.001*
White	97,298	5.5	5.3−5.7		4.7	4.4−5.0		6.5	6.2−6.8	
Black	24,849	5.5	5.1−6.0		4.3	3.9−4.8		7.3	6.7−7.9	
Hispanic	9,313	5.4	4.8−6.2		4.4	3.7−5.2		7.5	6.4−8.7	
Other^g^	5,646	7.1	6.4−8.0		6.1	5.2−7.2		8.9	7.7−10.2	
Not stated	16,412	4.4	3.7−5.2		3.8	3.1−4.7		5.1	4.4−5.8	
**Total days of hospital stay**				*<0.001*			*<0.001*			*<0.001*
<7 days	84,605	4.1	3.9−4.3		2.5	2.4−2.7		5.9	5.6−6.2	
≥7 days	68,913	7.1	6.7−7.4		6.8	6.3−7.2		7.6	7.1−8.0	
**Primary expected payer**				*<0.001*			*<0.001*			*<0.001*
Medicare	83,502	6.5	6.2−6.8		5.5	5.1−5.8		8.0	7.6−8.4	
Medicaid	16,014	4.7	4.3−5.2		3.6	3.2−4.1		6.4	5.7−7.1	
Private including HMO	41,706	4.0	3.7−4.3		3.4	3.1−3.8		4.5	4.2−4.9	
Self-pay or other payers	12,296	4.4	4.0−4.8		3.3	2.9−3.8		5.7	5.0−6.5	
**Operating room procedure**				*<0.001*			*<0.001*			*<0.001*
No procedure of any kind	60,751	1.8	1.6−1.9		1.4	1.2−1.5		2.2	2.0−2.4	
Non-operating room procedure^h^	50,369	9.1	8.7−9.5		7.2	6.8−7.7		12.0	11.3−12.7	
Operating room procedure^i^	42,398	6.3	6.0−6.7		5.3	4.9−5.7		8.1	7.5−8.6	
**Number of comorbidities** j				*<0.001*			*<0.001*			*<0.001*
0	10,540	1.5	1.3−1.8		1.6	1.3−2.0		1.4	1.1−1.8	
1	22,757	2.9	2.6−3.2		2.4	2.1−2.7		3.6	3.1−4.2	
2	30,779	4.1	3.8−4.4		3.4	3.0−3.8		4.9	4.5−5.4	
3	30,547	4.9	4.6−5.3		4.1	3.7−4.4		6.1	5.6−6.6	
4	24,421	6.5	6.1−6.9		5.7	5.2−6.2		7.4	6.8−8.0	
≥5	34,474	9.3	8.8−9.8		8.0	7.4−8.7		10.9	10.3−11.5	

a With a diagnosis of DVT only (without a diagnosis of PE).

b With a diagnosis PE regardless of DVT status.

c n = maximum unweighted subgroup size; N = Weighted population estimate.

d 95% confidence interval.

e *P*-value for Pearson Chi-Square test.

f May not be nationally representative because not all participating states collect race/ethnicity information.

g Includes categories of “other race/ethnicity,” “Native American,” and “Asian or Pacific Islander.”

h Had at least one minor diagnostic or minor therapeutic procedure (without any major diagnostic or major therapeutic procedure) during hospitalization.

i Had at least one major diagnostic or major therapeutic procedure during hospitalization.

j AHRQ 29-comorbidity index.

After adjusting for covariates including age, sex, race or ethnicity, total days of hospital stay, insurance status of primary expected payer, status of operating room procedure, and retained comorbidities, we found 13 of the AHRQ 29 comorbidities to be associated significantly and positively with in-hospital death among either hospitalizations with a diagnosis of VTE or among hospitalizations with a diagnosis of DVT or PE (Table 2). For example, in three separate regression models, when compared to the respective groups of hospitalizations with a diagnosis of VTE, DVT or PE that had no corresponding comorbidities, congestive heart failure, coagulopathy, liver disease, fluid and electrolyte disorders, metastatic cancer, peripheral vascular disorders, renal failure, solid tumor without metastasis, and weight loss were positively and independently associated with increased likelihoods of death (aORs) ranging from 1.3 (95% CI: 1.2−1.4) to 2.3 (95% CI: 2.1−2.4) times. In addition, chronic pulmonary disease was associated positively and independently with death in hospitalizations with VTE (aOR = 1.1; 95% CI: 1.0−1.2) or DVT (aOR = 1.2; 95% CI: 1.1−1.3); lymphoma and pulmonary circulation disorders were associated positively and independently with death in hospitalizations with VTE (aOR = 1.3; 95% CI: 1.2−1.5 and aOR = 1.2; 95% CI: 1.1−1.3, respectively) or in hospitalizations with a DVT (aOR = 1.6; 95% CI: 1.3−1.9 and aOR = 1.3; 95% CI: 1.1−1.6, respectively). Other neurological disorders also was associated positively and independently with death in hospitalizations with PE (aOR = 1.2; 95% CI: 1.0−1.3) (Table 2). The distribution patterns of the prevalence for having each of the 13 comorbidities in hospitalizations were similar across the three groups of hospitalizations with VTE, DVT, or PE and ranged from 1.4% for lymphoma to 31.9% for fluid and electrolyte disorder (Table 2). The c-statistic values that obtained from adjusted logistic regression and the ROC models ranged from 0.776 to 0.802. Whereas the c-statistic value obtained from logistic regression models with significant comorbidities suggested an excellent predictive accuracy for in-hospital death among hospitalizations with DVT, the values generally indicated an acceptable predictive accuracy for in-hospital death among hospitalizations with VTE and among hospitalizations with DVT (full model) or PE (Table 3).

**Table 2:** Prevalence of comorbidities and adjusted odds ratios for in-hospital death among adult hospitalizations with a diagnosis of VTE and among those with DVT or PE, NIS, 2010.

AHRQ 29-comorbidity index^a^		VTE(n = 153,518)					DVT^b^(n = 87,605)					PE^c^(n = 65,913)			
		Prevalence of comorbidity		In-hospital death		Prevalence of comorbidity			In-hospital death		Prevalence of comorbidity			In-hospital death	
	n^d^(153,518)	%	95% CI^e^	aOR^f^	95% CI	%		95% CI	aOR	95% CI	%		95% CI	aOR	95% CI
Acquired immune deficiency syndrome	553	0.4	0.3−0.4	−	−	0.4		0.3−0.5	−g	−	0.3		0.2−0.4	−	−
Alcohol abuse	5,131	3.4	3.2−3.6	0.8	0.7−1.0	3.5		3.2−3.7	0.8	0.6−0.9	3.2		3.0−3.5	−	−
Anemia (deficiency)	37,507	24.4	23.4−25.4	0.7	0.6−0.7	27.0		25.8−28.2	0.8	0.7−0.8	21.0		20.1−21.8	0.7	0.6−0.7
Arthritis (rheumatoid)/collagen vascular diseases	5,266	3.4	3.3−3.6	−	−	3.4		3.3−3.6	−	−	3.4		3.3−3.6	−	−
Chronic blood loss anemia	2,622	1.7	1.6−1.8	0.6	0.5−0.8	1.9		1.7−2.0	0.7	0.5−0.9	1.5		1.4−1.6	0.6	0.4−0.8
Congestive heart failure	19,465	12.7	12.2−13.1	**1.6**	**1.5−1.7**	12.5		12.0−13.0	**1.6**	**1.5−1.8**	12.9		12.4−13.4	**1.4**	**1.3−1.6**
Chronic pulmonary disease	31,939	20.7	20.1−21.4	**1.1**	**1.0−1.2**	18.7		18.0−19.4	**1.2**	**1.1−1.3**	23.5		22.7−24.2	−	−
Coagulopathy	13,359	8.7	8.2−9.2	**2.0**	**1.9−2.1**	9.5		8.9−10.2	**2.2**	**2.0−2.4**	7.7		7.3−8.1	**1.9**	**1.7−2.1**
Depression	16,506	10.7	10.3−11.1	0.8	0.7−0.8	10.4		9.9−10.8	0.7	0.7−0.8	11.1		10.7−11.6	0.8	0.7−0.9
Diabetes, uncomplicated	29,775	19.4	18.9−19.9	−	−	20.1		19.6−20.7	−	−	18.4		17.9−18.9	−	−
Diabetes with chronic complications	6,735	4.4	4.1−4.6	0.9	0.8−1.0	5.5		5.2−5.8	0.9	0.7−1.0	2.9		2.7−3.1	−	−
Drug abuse	4,447	2.9	2.6−3.2	0.6	0.5−0.7	3.0		2.7−3.3	0.5	0.4−0.7	2.8		2.5−3.1	0.6	0.5−0.8
Hypertension (uncomplicated and complicated)	83,680	54.5	53.5−55.5	0.7	0.7−0.7	55.1		54.0−56.2	0.7	0.7−0.8	53.7		52.7−54.6	0.7	0.6−0.7
Hypothyroidism	17,179	11.1	10.8−11.5	0.8	0.8−0.9	11.2		10.7−11.6	0.8	0.7−0.9	11.1		10.7−11.5	0.8	0.7−0.9
Liver disease	41,04	2.7	2.5−2.9	**1.5**	**1.3−1.7**	2.9		2.7−3.2	**1.8**	**1.5−2.0**	2.4		2.2−2.6	**1.3**	**1.0−1.5**
Lymphoma	2,521	1.6	1.5−1.8	**1.3**	**1.2−1.5**	1.8		1.7−1.9	**1.6**	**1.3−1.9**	1.4		1.3−1.6	−	−
Fluid and electrolyte disorders	44,984	29.3	28.3−30.3	**2.1**	**2.0−2.3**	31.9		30.7−33.1	**2.3**	**2.1−2.4**	25.9		25.1−26.7	**2.2**	**2.0−2.3**
Metastatic cancer	11,379	7.4	6.8−8.2	**2.1**	**2.0−2.3**	6.8		6.2−7.5	**2.1**	**1.9−2.4**	8.3		7.5−9.1	**2.0**	**1.8−2.2**
Other neurological disorders	14,522	9.4	9.2−9.7	−	−	10.3		9.9−10.7	−	−	8.3		8.0−8.6	**1.2**	**1.0−1.3**
Obesity	19,276	12.5	12.0−13.1	0.8	0.8−0.9	10.4		9.9−11.0	0.8	0.7−0.9	15.3		14.7−15.9	0.8	0.7−0.9
Paralysis	6,708	4.4	4.1−4.7	−	−	5.4		5.0−5.7	−	−	3.0		2.8−3.3	−	−
Peripheral vascular disorders	8,917	5.8	5.5−6.1	**1.3**	**1.2−1.4**	6.3		5.9−6.6	**1.3**	**1.1−1.4**	5.2		5.0−5.5	**1.3**	**1.1−1.4**
Psychoses	6,896	4.5	4.3−4.7	−	−	4.5		4.3−4.7	−	−	4.5		4.2−4.7	−	−
Pulmonary circulation disorders^h^	6,667	4.3	4.1−4.6	**1.2**	**1.1−1.3**	2.6		2.4−2.8	**1.3**	**1.1−1.6**	6.7		6.4−7.1	1.0	0.8−1.1
Renal failure	23,035	15.0	14.5−15.5	**1.4**	**1.3−1.5**	18.5		17.9−19.2	**1.3**	**1.2−1.4**	10.3		9.9−10.7	**1.6**	**1.4−1.7**
Solid tumor without metastasis	7,457	4.9	4.7−5.1	**1.4**	**1.3−1.5**	4.6		4.4−4.8	**1.4**	**1.2−1.6**	5.2		4.9−5.4	**1.3**	**1.1−1.5**
Peptic ulcer disease excluding bleeding	79	0.1	0−0.1	−	−	0.1		0−0.1	−	−	0		0−0.1	−	−
Valvular disease	6,733	4.4	4.1−4.6	0.9	0.8−1.0	3.8		3.6−4.1	−	−	5.1		4.8−5.5	0.8	0.7−1.0
Weight loss	15,385	10.0	9.2−10.9	**1.6**	**1.5−1.7**	11.8		10.7−12.9	**1.8**	**1.6−2.0**	7.7		7.1−8.3	**1.5**	**1.3−1.7**

a Referent groups were adult hospitalizations without corresponding comorbidity.

b With a diagnosis of DVT only.

c With a diagnosis PE with or without a diagnosis of DVT.

d Subgroup with comorbidity are shown (subgroups without comorbidity: n  = 153,518 - n for subgroup with corresponding comorbidity).

e Confidence interval.

f Odds ratios from logistic regression model with backward elimination procedure that adjusted for age (continuous), sex, race/ethnicity, total days of hospital stay (continuous), primary expected payer, major operating room procedure, and all comorbidities in the table without suppressed cells.

g Eliminated variable not in the final logistic regression model.

h Hospitalizations with PE and without other diseases in the category were classified as having negative status for the comorbidity.

Note: Comorbidities are significantly and positively associated with in-hospital death are bolded.

**Table 3:** Predictive accuracy of AHRQ 29-comorbidity index for in-hospital death among adult hospitalizations with a diagnosis of VTE and among those with DVT or PE, NIS, 2010.

	n	Comorbidity^a^	C-statistic^b^	95% CI^c^
**VTE**	n = 153,518			
		All 29 comorbidities	0.785	0.779− 0.791
		Selected comorbidities	0.785	0.779− 0.791
**DVT**	n = 87,605			
		All 29 comorbidities	0.779	0.771−0.787
		Selected comorbidities^d^	0.802	0.793−0.811
**PE**	n = 65,913			
		All 29 comorbidities	0.776	0.771−0.781
		Selected comorbidities	0.785	0.777−0.792

a Comorbidity retained in logistic regression model that also adjusted for age (continuous), sex, race/ethnicity, total days of hospital stay (continuous), primary expected payer, major operating room procedure.

b From logistic regression and ROC models with bootstrap replication estimation.

c 95% confidence interval.

d Selected comorbidities (unsuppressed) shown in Table 2.

## Discussion

By using a large, nationally representative sample, we observed that the AHRQ 29-comorbidity index has acceptable to excellent predictive accuracy for predicting death among hospitalizations with VTE and among hospitalizations with DVT or PE. Regardless of the diagnosis of DVT or PE, we found that 13 comorbidities−congestive heart failure, chronic pulmonary disease, coagulopathy, liver disease, lymphoma, fluid and electrolyte disorders, metastatic cancer, other neurological disorders, peripheral vascular disorders, pulmonary circulation disorders, renal failure, solid tumor without metastasis, and weight loss−each were associated independently with 10%−125% increased risk of in-hospital death. The predictive accuracy of the 29-comorbidity index and several nationally representative estimates from this study (e.g., the estimated number of deaths in hospitalizations with VTE and in hospitalizations with DVT or PE) have not been reported in the past.

The results of our study have a number of important implications for clinical and public health practice. First, health outcome such as mortality differs among patients and partly depends on their comorbidity status. Using the AHRQ 29-comorbidity index, our study provides several nationally representative estimates of mortality predictors that can aid in the counseling of patients with VTE and their families thereby contributing to evidence-based personalized prevention among high-risk patients. Second, in hospitalizations with VTE and hospitalizations with DVT or PE, the predictive accuracy remained similar, although we adjusted for only the 13 comorbidities that were associated positively and significantly with in-hospital death. Therefore, practitioners ought to be vigilant in assessing and identifying patients with VTE who have such comorbidities that might predispose them to heightened risks of death. Additionally, the potential clinical utilities of the 13 comorbidities as prognostic indicators for VTE may be further explored and developed in future research. Third, although the case-fatality rate for PE was higher than for DVT, the overall mortality burden related to hospitalizations with DVT (accounting for 20,212 deaths) was similar to those with PE (accounting for 21,732 deaths) in 2010, since the estimated number of hospitalizations with DVT was larger than that of hospitalizations with PE. Similarly for those deaths with DVT or PE, we found that a vast majority (about 7 in 10) of deaths with VTE had at least 3 other diseases concurrently. Given that major illnesses are associated with an increased risk of VTE in patients especially during times of immobilization [39], and that the presence of comorbidities is associated with a high-risk of death among hospitalizations with VTE, hospitalized patients with VTE affected by comorbidities are an important clinical and public health concern. Finally, besides being associated with worse health outcomes and complex clinical management, comorbidities potentially can alter both the efficacy of therapies and the course of the primary disease. With a growing population of aging adults in the United States, more research and clinical studies are needed that include patients with various, significant comorbidities so that optimal prevention strategies can be developed and intervention results can be extrapolated effectively for patients with VTE who have similar risk profiles.

Our study results were consistent with those of earlier studies that reported some comorbidities (e.g., congestive heart failure, liver disease, lymphoma, metastatic cancer, and renal failure) were associated independently with an increased risk of death among the general adult hospitalized populations [40]. Also consistent with findings of earlier studies, our study findings revealed that other comorbidities (e.g., anemia, depression, hypertension, obesity, and valvular disease) were associated with a decreased risk of in-hospital death which might reflect less urgent overall conditions of these patients [28], [40], [41]. On average, patients with Medicare as primary expected payer insurance were typically older than those with other types of health insurance. Not surprisingly, we observed a higher case-fatality rate in hospitalizations of patients with Medicare than that in subgroups with other types of health insurance. However, the exact reason for a lower case-fatality rate in hospitalization with “operating room procedure” than that with “non-operating room procedure” remained unknown, further research is needed to identify potential diagnostic and therapeutic procedures (e.g., venous catheterization and anticoagulation) or their associated conditions that may be responsible for an increased risk of in-hospital death. Nevertheless, our study was not without limitations. For instance, our findings were dependent on the accuracy of collected data (e.g., *ICD-9-CM* codes). Due to the incomplete reporting of race or ethnicity information by some states and potential misclassification, national estimates for race or ethnicity should be interpreted with caution. Because of the availability of data elements in the administrative datasets, we might not have controlled for all possible risk factors (e.g., anticoagulation) that otherwise might have affected the short-term risk of death during hospitalizations. Moreover, the NIS hospitalizations rates did not necessarily reflect rates per patient, as multiple hospitalizations of the same patients could have been included. Finally, the cross-sectional study design did not infer a causal relationship between risk factors and in-hospital death. Nevertheless, previous research suggested *ICD-9-CM* codes for VTE had 95% and 75% positive predictive values for the primary and secondary positions of diagnosis, respectively [42].

Comorbidity indices are useful for developing predictive models that can help clinicians to assess and identify high-risk patient populations, although they have not been adopted to predict the risk of individual patients. Currently, clinical information of patients is collected routinely in healthcare settings and yet clinical data are not always readily available to researchers and practitioners for instantaneous analysis, interpretation, and reporting. Additional research still is needed to explore, assess, develop, and integrate clinical and administrative data for real-time risk prediction and decision-support that might improve coordination, enhance evidence-based decision making, increase compliance, and achieve better health outcomes in healthcare systems. In conclusion, our results indicated that comorbidity was associated independently with risk of death among hospitalizations with VTE and among hospitalizations with DVT or PE. The AHRQ 29-comorbidity index provides acceptable to excellent predictive accuracy for in-hospital deaths among adult hospitalizations with VTE and among those with DVT or PE.
